# Efficient Treatment of Pulpitis via Transplantation of Human Pluripotent Stem Cell-Derived Pericytes Partially through *LTBP1*-Mediated T Cell Suppression

**DOI:** 10.3390/biomedicines11123199

**Published:** 2023-12-01

**Authors:** Anqi Li, Zhuoran Li, Weicheng Chiu, Chuanfeng Xiong, Qian Chen, Junhua Chen, Xingqiang Lai, Weiqiang Li, Qiong Ke, Jia Liu, Xinchun Zhang

**Affiliations:** 1 Hospital of Stomatology, Guanghua School of Stomatology, Sun Yat-sen University, Guangzhou 510055, China; lianq29@mail2.sysu.edu.cn (A.L.); chiuwch@mail3.sysu.edu.cn (W.C.); 2 Guangdong Provincial Key Laboratory of Stomatology, Guangzhou 510080, China; 3 Center for Stem Cell Biology and Tissue Engineering, Key Laboratory for Stem Cells and Tissue Engineering, Ministry of Education, Sun Yat-sen University, Guangzhou 510080, China; lizhr26@mail2.sysu.edu.cn (Z.L.); xcffimmu@163.com (C.X.); chenq297@mail2.sysu.edu.cn (Q.C.); chenjh526@mail2.sysu.edu.cn (J.C.); liweiq6@mail.sysu.edu.cn (W.L.); kqbear@163.com (Q.K.); 4 Department of Cardiology, The Eighth Affiliated Hospital, Sun Yat-sen University, Shenzhen 518033, China; laixq8@mail.sysu.edu.cn; 5 Guangdong Key Laboratory of Reproductive Medicine, Guangzhou 510080, China; 6 VIP Medical Service Center, The Third Affiliated Hospital, Sun Yat-sen University, Guangzhou 510630, China

**Keywords:** pericytes, pulpitis, neural crest, human pluripotent stem cells, *LTBP1*

## Abstract

Dental pulp pericytes are reported to have the capacity to generate odontoblasts and express multiple cytokines and chemokines that regulate the local immune microenvironment, thus participating in the repair of dental pulp injury in vivo. However, it has not yet been reported whether the transplantation of exogenous pericytes can effectively treat pulpitis, and the underlying molecular mechanism remains unknown. In this study, using a lineage-tracing mouse model, we showed that most dental pulp pericytes are derived from cranial neural crest. Then, we demonstrated that the ablation of pericytes could induce a pulpitis-like phenotype in uninfected dental pulp in mice, and we showed that the significant loss of pericytes occurs during pupal inflammation, implying that the transplantation of pericytes may help to restore dental pulp homeostasis during pulpitis. Subsequently, we successfully generated pericytes with immunomodulatory activity from human pluripotent stem cells through the intermediate stage of the cranial neural crest with a high level of efficiency. Most strikingly, for the first time we showed that, compared with the untreated pulpitis group, the transplantation of hPSC-derived pericytes could substantially inhibit vascular permeability (the extravascular deposition of fibrinogen, ** *p* < 0.01), alleviate pulpal inflammation (TCR^+^ cell infiltration, * *p* < 0.05), and promote the regeneration of dentin (** *p* < 0.01) in the mouse model of pulpitis. In addition, we discovered that the knockdown of latent transforming growth factor beta binding protein 1 (*LTBP1*) remarkably suppressed the immunoregulation ability of pericytes in vitro and compromised their in vivo regenerative potential in pulpitis. These results indicate that the transplantation of pericytes could efficiently rescue the aberrant phenotype of pulpal inflammation, which may be partially due to *LTBP1*-mediated T cell suppression.

## 1. Introduction

Pulpitis is a common inflammatory disease, and population-based studies worldwide have shown that about 48% of healthy individuals suffer from pulp injury [1]. Pulpitis is mainly caused by dental pulp bacterial infection, which is characterized by the local accumulation of inflammatory products in dental pulp [2], causing irreversible trauma to dental pulp and periapical tissue, and eventually leading to the loss of dental pulp function and tooth loss [3]. At present, the most common clinical treatments for pulpitis are root canal therapy (RCT) and vital pulp therapy (VPT). However, RCT may increase the risk of tooth fracture due to a lack of the nutritional and supportive effects of blood vessels and nerves. Reinfection in the crown edge and periodontal, secondary caries and leakage may occur after RCT, which could also eventually lead to tooth loss or extraction [1]. In addition, due to the limited efficacy of VPT treatment, there is a clinical trend towards the need to completely remove pulp tissue to control postoperative reinfection and pain [4]. Recent basic and clinical studies have shown that the transplantation of dental pulp stem cells can regenerate dental pulp in patients with pulpitis [5]. Therefore, the development of stem cell technology holds great promise in the provision of new therapeutic means for the treatment of pulpitis.

Dental pulp is a loose connective tissue containing abundant blood vessels and nerves to ensure the viability of teeth. Under normal physiological conditions, the pulp vasculature branches outward from the central vessel to form plexuses [6]. Pericytes are one of the key components of blood vessels, which wrap around endothelial cells (ECs) and perform diverse functions including the maintenance of vascular stability, the regulation of blood flow, barrier function, and immunomodulatory activity [7]. Nerve fibers in dental pulp also have an important role in the pathogenesis of pulpitis via mediating pain sensations, regulating blood flow, and modulating the inflammatory response [5,8,9,10]. When pulpitis occurs, sensory nerve fibers are activated by tissue hypoxia and pain and then secrete neuropeptides, which in turn lead to vasodilation and increased vascular permeability, and eventually result in pericyte death, vascular wall rupture, and other phenotypes [9,11]. It has been reported that endogenous dental pulp pericytes possess immunomodulatory properties [12] and can differentiate into odontoblasts and promote the repair of dental pulp injury in vivo [13]. These results strongly indicated the essential role of pericytes in dental pulp homeostasis, which prompt us to hypothesize that the restoration of pericytes may help to relieve the inflammation index and may represent a potential therapeutic option during pulpitis. However, it has not been reported whether the transplantation of exogenous pericytes can effectively treat pulpitis, and the underlying molecular mechanism remains unknown.

In this study, firstly, we showed that dental pulp pericytes originate from the neural crest through a lineage-tracing mouse model, *Wnt1-Cre2*, and the ablation of Pdgfrβ^+^ pericytes induced the leakage of dental pulp vasculature and disordered pulp and dentin structures. More importantly, we demonstrated for the first time that pericytes derived from human pluripotent stem cells (hPSCs) could effectively reduce vascular permeability, inhibit inflammation, and promote the formation of dentin in a mouse model of pulp infection. We also revealed that the knockdown of *LTBP1* significantly impaired the in vitro immunomodulatory property as well as in vivo tissue regeneration capacity of pericytes.

## 2. Materials and Methods

### 2.1. Animals

The mouse lines used in this study included *129S4.Cg-E2f1^Tg(Wnt1-cre)2Sor^/J* (*Wnt1-Cre2*, JAX:022137) [14], *Tg(Pdgfrβ-Cre)^35Vli^* (*Pdgfrβ-Cre*) [15], *B6.Cg-Gt(ROSA)26Sor^tm14(CAG-tdTomato)Hze^/J* (*Ai14*, JAX:007914) [16], *C57BL/6-Gt(ROSA)26Sor^tm1(HBEGF)Awai^/J* (*ROSA26iDTR*, JAX:007900) [17], and wild-type *C57BL/6J* (Beijing Vital River Laboratory Animal Technology Co., Ltd., Beijing, China). The mice were maintained in a specific-pathogen-free facility. All experimental procedures involving animals were approved by the ethics committee of Hospital of Stomatology, Guanghua School of Stomatology, Sun Yat-sen University.

### 2.2. Induction of Pulpitis

Ninety-three *C57BL/6* mice (6–8 weeks old) weighing 20–30 g were randomly divided into a control group (thirty-three mice) and experimental group (for pulpitis modeling; sixty mice) and anesthetized via the intraperitoneal (i.p.) administration of pentobarbital sodium (50 mg/kg). The pulpitis was induced as previously reported [18]. In brief, a class 1 cavity was meticulously prepared on the occlusal surface of the bilateral mandibular first molars using a #1/4 dental round bur, the whole procedure for which was carried out while utilizing a stereomicroscope. The mandibular first molar was carefully drilled at medium speed with a water-cooling system until the pulp became visible through the transparency of the dentin on the cavity floor. Thereafter, the pulp was exposed using a specialized endodontic hand file, featuring a 0.15 mm diameter tip with a 2% taper and a length of 21 mm. The exposed preparation cavity was left open to the oral environment. Twenty-four hours later, human pluripotent stem cell (hPSC)-derived pericytes of cranial neural crest origin (CNC PCs) were transplanted into the pulp cavities of twenty-four randomly selected pulpitis model mice through local injection.

### 2.3. Diphtheria Toxin-Dependent Ablation of Pericytes

To generate the inducible pericyte ablation model, we crossed *Pdgfrβ-Cre* mice with *ROSA26-iDTR* mice for the Cre-dependent expression of DTR and generated *Pdgfrβ-Cre;iDTR* mice. Males and females from both lines were used for breeding and to maintain the colony. Two- to three-month-old *Pdgfrβ-Cre;iDTR* mice were administered i.p. 0.1 µg of diphtheria toxin (DT; Sigma-Aldrich, St. Louis, MO, USA, *n* = 15) or a vehicle (*n* = 15) every day for 10 consecutive days, as previously reported [19].

### 2.4. Pericytes Derived from Human Pluripotent Stem Cells

An H1 embryonic stem cell line (obtained from WiCell Research Institute [20]) and a human-induced pluripotent stem cell line (hiPSCs) generated in our laboratory from human embryonic fibroblasts using retroviral vectors expressing *OCT4*, *KLF4*, *SOX2*, and *c-MYC* [21] were used in this study. Undifferentiated hPSCs were cultured on Matrigel (BD Biosciences, San Diego, CA, USA)-coated dishes in mTeSR1 medium (StemCell Technologies, Vancouver, BC, Canada) in a humidified incubator with 5% CO_2_ at 37 °C. The medium was changed every day, and confluent cells were passaged at a 1:6 ratio using ReLeSR™ (StemCell Technologies).

For pericyte differentiation, hPSCs or DsRedE2-hPSCs were first differentiated to the cranial neural crest followed by pericyte commitment, as previously described [22]. Confluent hPSCs were first dissociated into single cells using Accutase and then replated onto Matrigel-coated plates with a density of 2 × 10^4^ cells/cm^2^. The cells were cultured in a completely defined medium (CDM) consisting of DMEM-F12 as the basal medium supplemented with 1 × N2, 1 × B27, 1 mM L-glutamine, 0.1 mM 2-mercaptoethanol, 1% MEM nonessential amino acid solution (all from Thermo Fisher Scientific, Rutherford, NJ, USA), 20 ng/mL of basic fibroblast growth factor (bFGF; PeproTech, Rocky Hill, NJ, USA), and 10 μM of Y-27632 (Sigma, St. Louis, MO, USA) for 24 h (D-1-D0). Then, the medium was replaced by an NCN2 medium composed of DMEM-F12, 1 × N2, 1.0 μM of CHIR99021, and 0.5 μM of SB431542 for 7 days. Then, the cells were dissociated and labeled with antibodies against HNK1 and low-affinity nerve growth factor receptor (NGFR, also known as p75), both from BD-Pharmingen (Palo Alto, CA, USA), for fluorescence-activated cell sorting (FACS) using a BD Influx cell sorter (BD-Pharmingen). p75^high^/HNK1^+^ cranial neural crest stem cells (CNCs) were enriched and cultured in neural crest culture medium (NCCM) containing DMEM-F12, 1 × N2, 1 × B27, 10 μM of Y27632, 20 ng/mL of bFGF, and 20 ng/mL of epidermal growth factor (EGF; PeproTech) at a density of 5 × 10^4^ cells/cm^2^. Twenty-four hours later, the medium was changed to pericyte medium (ScienCell Research Laboratories, Carlsbad, CA, USA) supplemented with 10 ng/mL of bFGF and 50 ng/mL of PDGFBB for 14 days to generate cranial neural crest-derived pericytes (CNC PCs).

### 2.5. Sample Preparation

Mice were sacrificed 24 h after pulpal exposure or 48 h after cell transplantation. Three mandibles with pulpal exposure without cell implantation were carefully dissected free of soft tissues, subsequently fixed in 4% paraformaldehyde (PFA) at 4 °C for 48 h, replaced with 70% ethanol, and then imaged using microcomputed tomography (micro-CT; SCANCO µCT100, ZÜRICH, Brüttisellen, Switzerland) imaging, operated at 90 kV and 200 µA as reported [18]. The remaining samples were used for subsequent hematoxylin and eosin (H&E) staining (*n* = 6), FACS analysis (*n* = 9), immunofluorescence staining (*n* = 42), and QRT-PCR testing (*n* = 21). All sample sizes are indicated in figure legends.

### 2.6. Dextran Assay

For the in vivo dextran assay, the tracer, fluorescein isothiocyanate (FITC)-tagged dextran (FITC-dextran, MW = 4000 Da; Sigma), was injected intravenously via the tail vein and allowed to circulate 1 h before scarification in the control group (*n* = 6) and the pulpal exposure group (*n* = 6). Then, dental pulp samples were collected, chopped, and placed in 1 mL of PBS. The liberated contents were homogenized for 45 s, and tissue and coarse particles were removed via centrifugation (300× *g*, 3 min). The supernatant was used to measure the fluorescence intensity of FITC-dextran using a Tecan plate reader with excitation at 485 nm and emission at 528 nm according to the manufacturer’s instructions.

In the in vitro dextran assay (transcytosis assay) [22,23], CNC PCs were cocultured with human umbilical vein endothelial cells (HUVECs) within 12-well Transwell inserts (8 μm pore size membrane; Corning, NY, USA) that had been precoated with collagen (1 μg/mL; Sigma) and fibronectin (10 μg/mL; Sigma) for a minimum of 4 h at 37 °C. CNC PCs (3 × 10^4^ cells/cm^2^) were initially seeded on the lower surface of the inserts and incubated at 37 °C in 5% CO_2_ for 2 h to ensure strong adherence (*n* = 3). Subsequently, HUVECs were loaded onto the upper surface of the inserts at a density of 3 × 10^5^ cells/cm^2^. FITC-dextran was added to the upper (luminal) chamber at a final concentration of 0.5 mg/mL. The fluorescence (485 nm excitation/528 nm emission) was determined via the collection of 100 μL aliquots in triplicate from each lower (abluminal) chamber every 30 min using a Tecan plate reader.

### 2.7. Tube Formation Assay

In each well of a 24-well plate, 200 μL of growth factor-reduced Matrigel (diluted 1:1 with cold DMEM on ice; BD Biosciences) was dispensed and allowed to solidify for 30 min at 37 °C as described [24]. Then, 5 × 10^4^ HUVECs (EC group, *n* = 3) or 5 × 10^4^ HUVECs with 1 × 10^4^ CNC PCs (EC + CNC PCs group, *n* = 3) were plated onto each well. The cells were observed under a bright-field microscope after incubation at 37 °C in 5% CO_2_ for 6 and 12 h. Tube formation was evaluated by quantifying tube lengths, nodes, junctions, and branches using the Angiogenesis Analyzer module in the ImageJ toolkit, as previously reported [25,26].

### 2.8. Multilineage Differentiation of CNC PCs

To initiate osteogenic differentiation [27], 1 × 10^5^ CNC PCs were seeded in one well of six-well plates and cultured in osteogenic medium supplemented with 100 μM of dexamethasone, 50 mM ascorbate-2-phosphate, 10 mM b-glycerophosphate (all from Sigma), and 1% (*v*/*v*) penicillin/streptomycin (Thermo Fisher Scientific) for 21 days. The culture medium was changed every 2–3 days. After 21 days, Alizarin Red S staining was performed to evaluate the osteogenic differentiation capacity of CNC PCs.

For the chondrogenesis assay [27], 5 × 10^5^ CNC PCs were seeded in a 15 mL centrifuge tube to form a pellet via centrifugation and incubated in commercial chondrogenic medium (StemCell Technologies, Vancouver, BC, Canada) for a duration of 3–4 weeks. Then, the chondrogenic differentiation potential of CNC PCs was determined through Alcian blue staining.

To promote the adipogenic differentiation [27], 1 × 10^5^ CNC PCs were plated onto one well of six-well plates and cultivated in adipogenic medium containing 500 μM of isobutyl-methylxanthine (Sigma), 1 mM dexamethasone, 10 mM insulin (Sigma), 200 mM indomethacin (Sigma), and 1% (*v*/*v*) penicillin/streptomycin (Thermo Fisher Scientific) for 14–21 days. The medium was refreshed every 2 days. The adipogenic differentiation capability of CNC PCs was evaluated via Oil Red O staining.

### 2.9. Detection of Immunoregulation Ability of CNC PCs

The influence of pericytes on the proliferation of CD3^+^ T lymphocytes was determined using a coculture method [28]. CD3^+^ T cells were obtained from healthy donors with fully informed consent, and the lymphocytes were enriched via FACS. All procedures performed in studies involving human participants were conducted in accordance with the standards of the ethics committee of Sun Yat-sen University and with the 1964 Declaration of Helsinki and its later amendments or comparable ethical standards.

The lymphocytes that were stained with carboxyfluorescein succinimidyl ester (CFSE) were cocultured with (*n* = 4) or without (*n* = 4) pericytes and stimulated with anti-CD3 mAb and anti-CD28 mAb (both from BD-Pharmingen) for 96 h. Then, the proliferation of CD3^+^ T lymphocytes was examined via FACS.

Isolated CD3^+^ T cells were cultured with (*n* = 3) or without (*n* = 3) pericytes for 72 h. Thereafter, cells were cultured in a medium supplemented with 10 μg/mL of brefeldin A (BFA), 50 ng/mL of phorbol-12-myristate-13-acetate (PMA), and 1 μg/mL of ionomycin (all from Sigma-Aldrich) for an additional 6 h. The expression levels of cytoplasmic TNF-α and IFN-γ in CD3^+^ cells were determined using flow cytometry.

### 2.10. Construction and Transduction of Short-Hairpin RNA (shRNA) Vector

Two shRNA sequences targeting human *LTBP1* were designed and separately introduced into linearized lentiviral vector pLL3.7 containing a dTomato-coding sequence (obtained from VectorBuilder, Guangzhou, China). To package lentivirus for the generation of stable cell lines, 293FT cells were cotransfected with each recombinant pLL3.7 vector and lentiviral packaging mix. Next, 72 h after transfection, the viral supernatant was collected and centrifuged at 50,000× *g* for 90 min to remove cellular debris and filtered through a 0.22 μm PES membrane filter (Merck, Darmstadt, Germany). The supernatant was used for the transduction of pericytes. dTomato-positive cells were then isolated via flow cytometry and expanded in vitro, and the mRNA and protein expression levels of LTBP1 were detected. A nontarget vector was used as a negative control (NTC).

### 2.11. Histologic Analysis

Teeth were rinsed, fixed in 4% paraformaldehyde at 4 °C, decalcified with 10% EDTA for 4 weeks, dehydrated, and embedded in paraffin; 4 mm thick serial sections were cut using a cryomicrotome (RWD FS800, Shenzhen, China) in the mesiodistal direction and used for histologic analyses. Samples were analyzed via hematoxylin and eosin (H&E) staining (*n* = 6) and immunostaining (*n* = 42).

### 2.12. Quantitative Reverse Transcription–Polymerase Chain Reaction (QRT-PCR)

QRT-PCR was performed as described previously [27]. Firstly, the total RNA was extracted from cells or tissues using TRIzol Reagent (Thermo Fisher Scientific) in accordance with the manufacturer’s recommendations. Subsequently, the RNA was reverse transcribed into cDNA using the Superscript II reverse transcriptase enzyme (Thermo Fisher Scientific). QRT-PCR was carried out using a LightCycler 480 SYBR Green I Master (Roche Diagnostics, Mannheim, Germany) on a LightCycler 480 Detection System (Roche Diagnostics). The PCR program consisted of 40 cycles with denaturation at 95 °C (10 s), annealing at 60 °C (20 s), and extension at 72 °C (30 s). *GAPDH* was utilized as an internal reference, and gene expression levels were determined using the 2^−ΔΔCT^ method. The detailed primer sequences are listed in Supplementary Table S1.

### 2.13. Immunocytochemistry

For immunocytochemistry [27], samples were fixed with 4% PFA for 20 min, followed by blocking with blocking buffer containing 0.3% Triton X-100 and 5% human BSA for 1 h. Subsequently, primary antibody incubation (the appropriate antibody concentration with 5% human BSA) was performed at 4 °C overnight. Afterward, the samples were washed three times with 1× PBS for 5 min and then incubated with the fluorochrome-conjugated secondary antibody in a dark, humidified chamber at room temperature for 1 h. To visualize the nucleus, 4′, 6-diamino-2-phenylindole (DAPI; Sigma-Aldrich) was used, and the results were photographed and analyzed using a laser scanning confocal microscope (LSM 880; Airyscan, Zeiss, Oberkochen Baden, Germany). At least three replicates of the experiment were carried out for each set of samples to be stained. The antibodies used for immunocytochemistry are presented in Supplementary Table S2.

### 2.14. Fluorescence-Activated Cell Sorting (FACS) Analysis

FACS was used to detect the protein expression of the surface antigens (including CD13, CD146, NG2, PDGFRβ, HNK1, and p75) (all from BD Biosciences) in pericytes or the cranial neural crest. An irrelevant isotype-identical antibody (BD Biosciences) was used as a negative control.

For in vitro cultured hPSC derivatives, cells were digested using Accutase, filtered, and incubated with anti-human monoclonal antibodies. To obtain the tooth samples from the mice, the animals were anesthetized and transcardially perfused with precooled PBS followed by 4% paraformaldehyde. The mandibular first molar was isolated and the surrounding soft tissues were removed (*n* = 9). Teeth were cut up and incubated in type I collagenase on a shaker at 37 °C for 4 h. Then, the sample was filtered through a 70 μm filter and rinsed with precooled PBS at 4 °C. After centrifugation at 1500 rpm for 5 min, the precipitate was resuspended in PBS for FACS analysis.

FACS was performed using a Beckman Coulter flow cytometer, and the data were analyzed with FlowJo software v10.8.1 (BD Biosciences) or CytExpert (Beckman Coulter GmbH, Krefeld, Germany). The antibodies utilized for FACS are listed in Supplementary Table S3.

### 2.15. Statistical Analysis

All experiments carried out in this study were replicated at least three times. All results are depicted as the mean ± standard deviation (SD) based on a minimum of three independent experiments. Differences between groups were tested via independent sample *t* tests and one-way analyses of variance (ANOVAs) with a post hoc test using the Student–Newman–Keuls test. Data comparison between two groups such as QRT-PCR, flow cytometry, mean fluorescence intensity, and proportion of positive cells was performed using *t*-test. One-way ANOVA was used for data comparison among three groups such as QRT-PCR and flow cytometry. All data were analyzed using GraphPad Prism 7 software and are presented as the mean plus standard deviation. In all analyses, a two-sided *p* value < 0.05 was regarded as statistically significant (95% CI).

## 3. Results

### 3.1. Pdgfrβ Is a Specific Marker of Dental Pulp Pericytes

**Figure 1:**
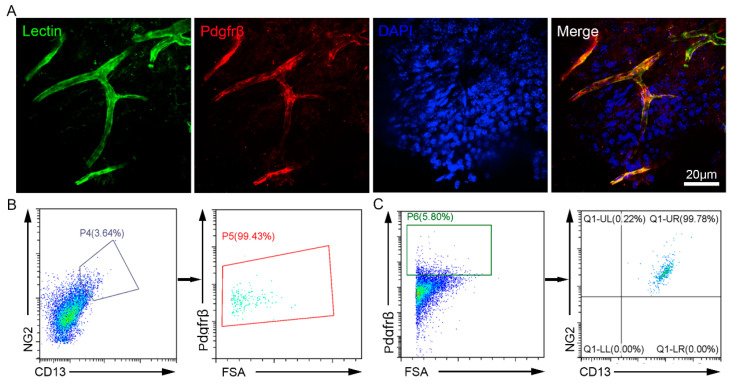
Detection of Pdgfrβ expression in dental pulp. (**A**) Immunofluorescence staining of Lycopersicon Esculentum (Tomato) Lectin (green) and Pdgfrβ (red) in dental pulp tissue (Dragonfly CR-DFLY-202 2540; 63 × 10 magnification). Scale bar: 20 μm. (**B**) FACS analysis for the expression of Pdgfrβ in NG2^+^/CD13^+^ cells (Beckman Coulter CytoFLEX). (**C**) FACS analysis for the expression of NG2 and CD13 in Pdgfrβ^+^ cells (Beckman Coulter CytoFLEX).

To verify whether Pdgfrβ can be used as a specific marker of pulp pericytes, we performed immunofluorescence staining and flow cytometry to verify whether Pdgfrβ^+^ cells are indeed pericytes in the dental pulp of wild-type C57 mice. The results of immunofluorescence staining showed that most of the Pdgfrβ^+^ cells localized near the lectin-Dylight488^+^ endothelial cells (Figure 1A). For FACS analysis, entire mouse teeth were digested, and we found that about 3–4% of the total cells coexpressed the pericyte markers CD13 and NG2. Moreover, the results showed that Pdgfrβ was expressed in almost all (>99%) of the CD13/NG2 double-positive cells (Figure 1B), and Pdgfrβ-expressing cells were also homogeneously positive for both NG2 and CD13 expression (>99%; Figure 1C). These results demonstrated that Pdgfrβ could be used as a suitable marker for labeling dental pulp pericytes.

### 3.2. Dental Pulp Pericytes Are Derived from Cranial Neural Crests

Previous studies indicated that dental pulp pericytes might originate from cranial neural crest cells [29]. To verify this hypothesis, we used a *Wnt1-cre2:Rosa26-tdTomato* mouse model to identify the developmental origin of Pdgfrβ^+^ pericytes. Immunofluorescence staining showed that, although most of the cells in dental pulp were tdTomato^+^, only a small population expressed Pdgfrβ. More importantly, Pdgfrβ-expressing pericytes were found to be homogeneously positive for tdTomato (Figure S1A). Flow cytometry also unveiled that tdTomato was expressed in most of the Pdgfrβ^+^ pericytes (>99%), while about 20% of tdTomato-expressing cells were labeled with the anti-Pdgfrβ antibody (Figure S1B,C). These results further confirmed that the pericytes in mouse dental pulp are derived from cranial neural crests.

### 3.3. Ablation of Endogenous Pdgfrβ^+^ Pericytes Induces Pulpitis-like Phenotypes

**Figure 2:**
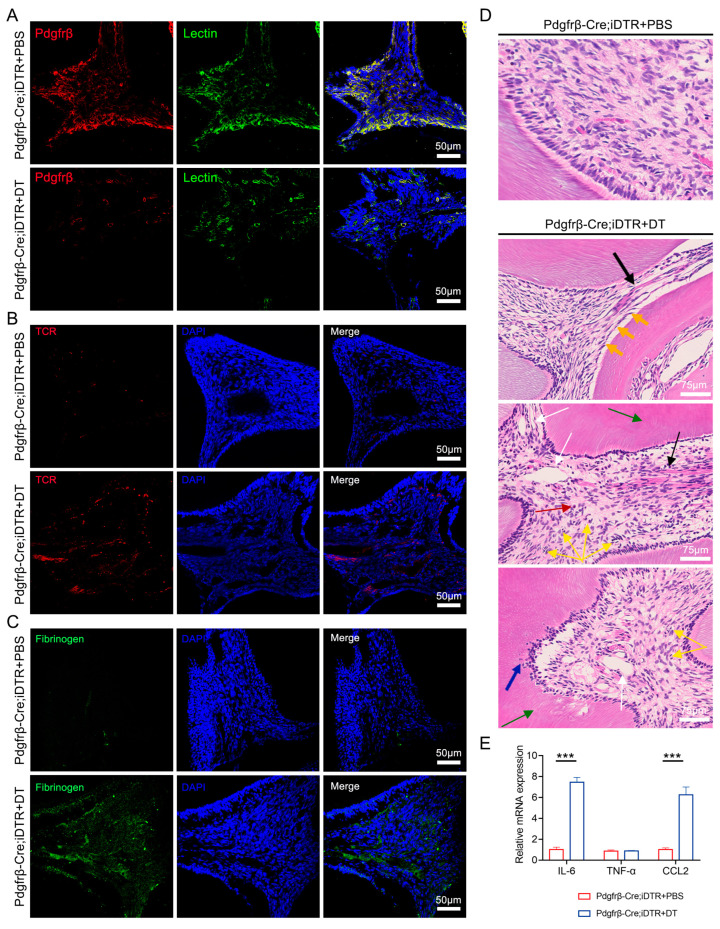
Detection of the changes in dental pulp after ablation of endogenous Pdgfrβ^+^ pericytes by DT. (**A**) Immunofluorescence staining of Lycopersicon Esculentum (Tomato) Lectin (green) and Pdgfrβ (red) in control group and DT-treated group (LSM 880 with Airyscan; 20 × 10 magnification). Scale bar: 50 μm. (**B**) Immunofluorescence staining of TCR (red) in control group and DT-treated group (LSM 880 with Airyscan; 20 × 10 magnification). Scale bar: 50 μm. (**C**) Immunofluorescence staining of fibrinogen deposits (green) in control group and DT-treated group (LSM 880 with Airyscan; 20 × 10 magnification). Scale bar: 50 μm. (**D**) H&E staining for mice dental pulp in control group and DT-treated group. Ablation of endogenous PDGFRβ^+^ pericytes induced pulpitis-like pathology, such as vascular structure abnormality (black arrows), vacuolar degeneration of the odontoblastic layer (orange arrows), abnormal curvature of dentinal tubules (green arrows), vasodilation (white arrows), osteodentin (blue arrows), and infiltration of granulocytes (yellow arrows) plasma cells (red arrows) (OLYMPUS BX51; 20 × 10 magnification). Scale bar: 75 μm. (**E**) Relative mRNA expression of *IL-6*, *TNF-α*, and *CCL2* in control group and DT-treated group; *n* = 3. The data in each panel represent the means ± SD; *p* values were obtained by Student’s two-tailed unpaired *t* test: *** *p* < 0.001.

To explore the role of pericytes in the homeostasis of dental pulp, we tried to eliminate Pdgfrβ^+^ pericytes and then detect the structural changes and inflammation index in dental pulp. The *Pdgfrβ-Cre* model was crossed with the *ROSA26-iDTR* line to construct *Pdgfrβ-Cre;iDTR* mice, which were then treated with diphtheria toxin (DT) for 10 consecutive days to ablate Pdgfrβ^+^ pericytes. An immunofluorescence assay confirmed that Pdgfrβ^+^ cells were significantly reduced after DT treatment compared with those in the PBS group (Figure 2A). To determine whether pericyte ablation results in the alteration of vascular permeability, we detected the presence of immune cells and the extravascular deposition of fibrinogen in dental pulp. The results showed that TCR^+^ cells and fibrinogen deposits substantially increased after DT treatment compared with those in the control group (Figure 2B,C), indicating a vascular leakage phenotype due to the loss of pericytes. Furthermore, H&E staining showed evidence of vascular structure abnormalities, osteodentin, the vacuolar degeneration of the odontoblastic layer, abnormal curvatures of dentinal tubules, and the infiltration of granulocytes and plasma cells in the pulp tissue after DT treatment, while these phenotypes were barely detected in the control group (Figure 2D). We also collected the mRNA of pulp tissue and performed QRT-PCR to analyze the expression of inflammatory cytokines that were highly upregulated in pulpitis [9]. The results suggested that the mRNA levels of *TNF-α* remained unchanged, while the transcripts of *IL-6* and *CCL2* were strikingly higher in the DT-treated group compared to in the control group (Figure 2E). This evidence suggests that the loss of the pericyte population could lead to a pulpitis-like phenotype, indicating the indispensable role of pericytes in the maintenance of homeostasis in dental pulp.

### 3.4. Impairment of Pericytes Was Detected in Mouse Model of Pulpitis

**Figure 3:**
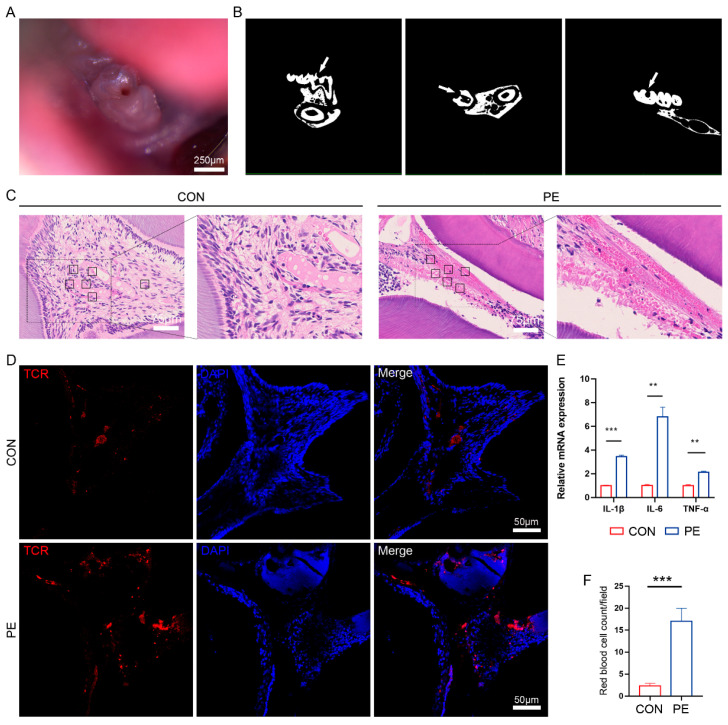
Establishment of the mice pulpitis model. (**A**) Pulp exposure image of the mandibular first molar by stereomicroscope (Leica MZ10F; 2.0 × 10 magnification). Scale bar: 250 μm. (**B**) The sagittal, transverse, and coronal micro-CT images of the mandibular first molar (arrows indicate a pulp lesion). (**C**) H&E staining of dental pulp from control group and pulpal exposure (PE) group (OLYMPUS BX51; 20 × 10 magnification). Scale bar: 75 μm. (**D**) Immunofluorescence staining of TCR (red) in control group and pulpal exposure (PE) group (LSM 880 with Airyscan; 20 × 10 magnification). Scale bar: 50 μm. (**E**) Relative mRNA expression of *IL-1β*, *IL-6*, and *TNF-α* in control group and pulpal exposure (PE) group; *n* = 3. (**F**) The average number of red blood cells in control group and pulpal exposure (PE) group in panel C was counted and compared (black square frames in panel C indicate six randomly selected fields of view for red blood cell counting); *n* = 6. The data in each panel represent the means ± SD; *p* values were obtained by Student’s two-tailed unpaired *t* test: ** *p* < 0.01, *** *p* < 0.001.

To determine whether the number and function of dental pulp pericytes change in response to bacterial infection, a convenient mouse model, experimental pulpal exposure (PE), was set up according to the previous report [18] to recapitulate pulp inflammation. The exposed preparation cavity in the mandibular first molar was clearly observed under a stereomicroscope, and the walls of the teeth remained intact (Figure 3A). The pulp and periapical area were then detected via a Micro-CT assay, and the images illustrated that the dental pup was directly exposed to the oral environment. Moreover, the size of the pulpal exposure area stayed approximately the same, without crowns or root fractures in the sagittal, transverse, or coronal directions (Figure 3B). No obvious periapical shadows or alveolar bone resorptions were observed 72 h after pulpal exposure.

To ascertain whether the pulpitis was successfully induced, we collected samples 24 h after pulpal exposure. H&E staining demonstrated that the control (CON) group maintained pulp tissue integrity and a regularly arranged odontoblast cell layer without evidence of inflammatory cell infiltration. However, we observed disordered odontoblast cell layers, sparsely arranged dentinal tubules with abnormal curvatures, and obvious inflammatory cell infiltration after pulpal exposure (PE group; Figure 3C). Additionally, anti-TCR immunofluorescence staining revealed that the proportion of TCR^+^ cells was dramatically upregulated in the experimental group compared with that in the control sample (Figure 3D). The mRNA expressions of the inflammatory factors, including *IL-1β*, *IL-6*, and *TNF-α*, in dental pulp were also dramatically increased after pulpal exposure, as detected during QRT-PCR (Figure 3E). These data suggest that the mouse model of pulpitis was successfully established.

**Figure 4:**
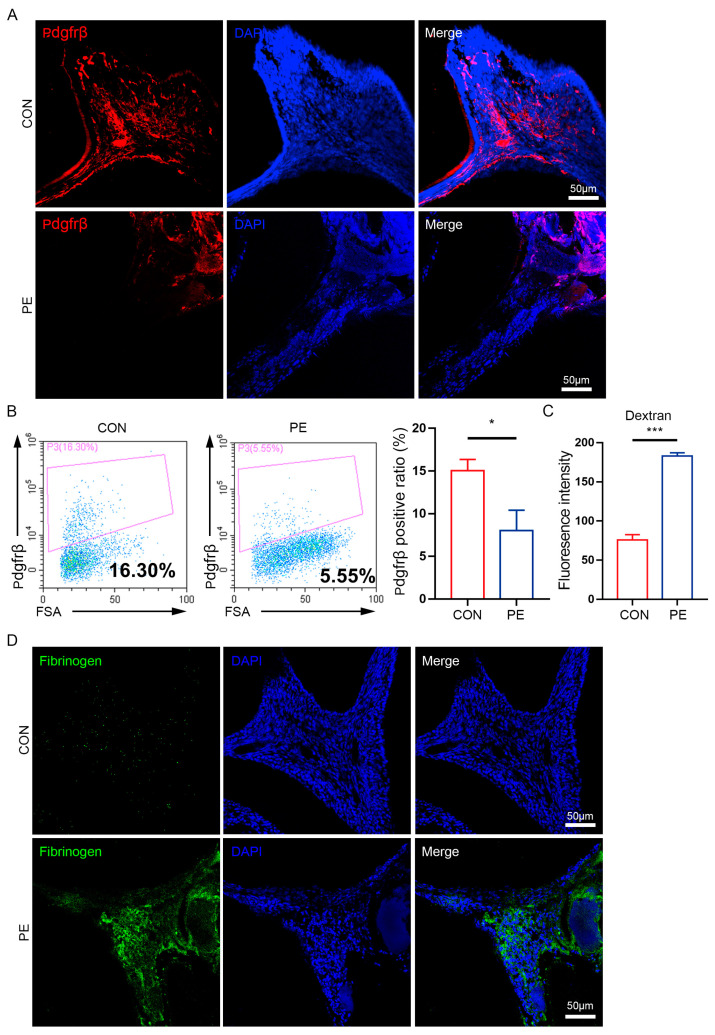
Decrease in pericytes and the increase in vascular leakage were detected during pulpitis. (**A**) Immunofluorescence staining of Pdgfrβ (red) in control group and pulpal exposure (PE) group (LSM 880 with Airyscan; 20 × 10 magnification). Scale bar: 50 μm. (**B**) FACS analysis (Beckman Coulter CytoFLEX) of Pdgfrβ^+^ pericytes in control group and pulpal exposure (PE) group (left panel) and the proportion of Pdgfrβ^+^ cells is counted and compared between control group and pulpal exposure (PE) group (right panel); *n* = 3. (**C**) The fluorescence intensity of Dextran was detected in control group and pulpal exposure (PE) group; *n* = 6. (**D**) Immunofluorescence staining of fibrinogen deposits (green) in control group and pulpal exposure (PE) group (LSM 880 with Airyscan; 20 × 10 magnification). Scale bar: 50 μm. The data in each panel represent the means ± SD; *p* values were obtained by Student’s two-tailed unpaired *t* test: * *p* < 0.05, *** *p* < 0.001.

In addition, we noticed the obvious extravasation of red blood cells into pulp tissue in the experimental group, and the number of red blood cells was 5- to 6-fold higher in the pulpitis group (17.0 ± 7.32 per field) than the control group (2.3 ± 1.51 per field) (Figure 3C,F), indicating an increase in vascular permeability after pulpal exposure. More importantly, immunofluorescence staining showed that there were noticeably fewer Pdgfrβ^+^ pericytes in the experimental group than in the control group (Figure 4A). FACS analysis also highlighted a reduction in the number of pericytes in pulpitis (CON group: 15.0 ± 1.32%; PE group: 8.0 ± 2.40%) (Figure 4B), which was consistent with the results from immunofluorescence staining. Then, an FITC-dextran assay was performed, and the exogenous leakage of dextran was detected in the pulp tissue supernatant via fluorospectrometry. Our results showed that the absorbance (OD) values increased tremendously in the experimental group (183.1 ± 4.73) compared with the control group (74.4 ± 9.87) (Figure 4C). Additionally, the detection of the extravascular deposition of fibrinogen via anti-fibrinogen staining also showed similar results to the dextran assay (Figure 4D), further confirming the vascular leakage phenotype in pulpitis. This evidence demonstrated that pulp inflammation could lead to the impairment of pericytes and the destruction of the structural integrity of vascular walls in dental pulp.

### 3.5. Pericyte Numbers in Human Dental Pulp Are Significantly Reduced in Deep Dentine Caries

To investigate whether dental pulp pericyte loss would occur in the presence of bacterial infection in caries or pulpitis in humans, we reanalyzed the reported single-cell RNA sequencing data of human dental pulp from normal teeth, enamel caries, and deep dentine caries [30], and tried to identify the cell subpopulations in these samples. Cell-type labeling was performed using the expression of lineage-specific markers (Figure S2A). The results showed that cell types and their numbers in the dental pulp tissue of enamel caries were similar to that in normal teeth, but they were obviously different from the deep dental caries group. A total of 14 cell populations were identified in the dental pulp with normal and enamel caries: OMD^+^ odontoblasts (ODBs), early ODBs, ALPL^+^ ODBs, DMP1^+^ ODBs, macrophages, pericytes, T cells, plasma cells, endothelial cells, hematopoietic stem cells (HSCs), NK cells, glial cells, Schwann cells, and CD16^+^ monocytes. Interestingly, 16 cell populations, including B cells and CD103^+^ dendritic cells (DCs), were identified in the teeth with deep caries (Figure S2B). Our results also revealed that B cells and CD103^+^ DCs were only found in teeth with deep caries (for B cells, 5.38% in deep caries; for CD103^+^ DCs, 0.63% in deep caries), while plasma cells were much more abundant in deep caries compared to in sound teeth and teeth with enamel caries (2.64% in deep caries, 0% in enamel caries, 0.22% in sound teeth) (Figure S2B). These results were in accordance with a previous report [30]. More importantly, we found that the proportion of pericytes decreased significantly in deep caries compared with that in sound teeth and enamel caries (3.73% in deep caries, 7.2953% in enamel caries, 7.3034% in sound teeth) (Figure S2C). These results further indicate a reduction in dental pulp pericytes in the presence of bacterial infection in humans.

### 3.6. Pericytes Are Successfully Generated from hPSCs through the Intermediate Stage of Cranial Neural Crests

**Figure 5:**
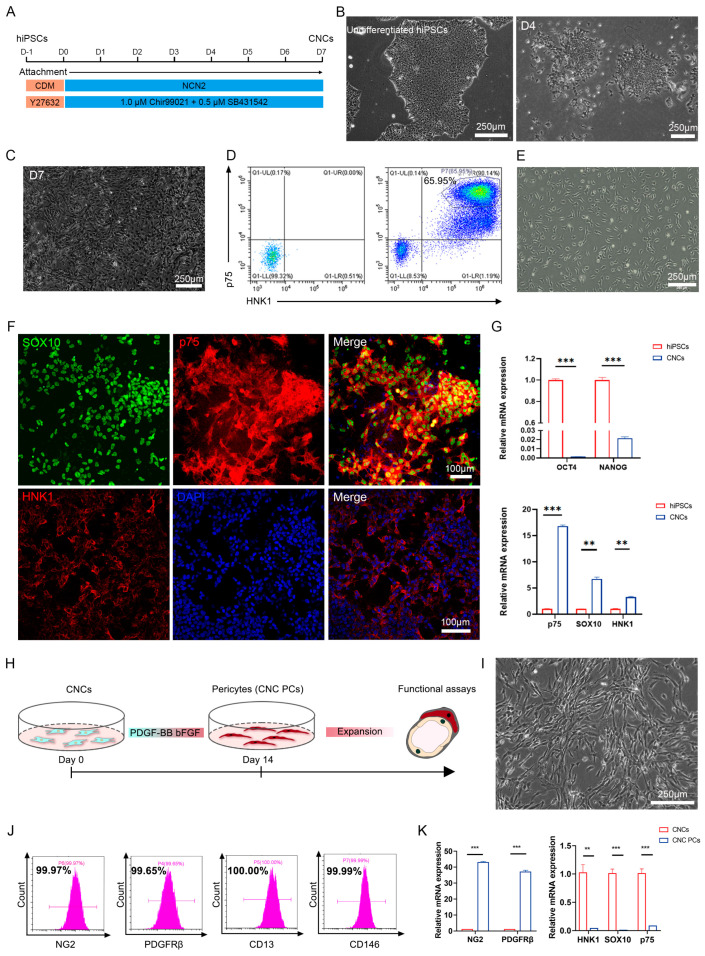
Differentiation of pericytes from human pluripotent stem cells. (**A**) Strategy for differentiation of cranial neural crest from hPSCs in monolayer cultures. (**B**) The morphology of undifferentiated hPSCs and day 4 (D4) differentiated cells was observed using phase-contrast microscopy (Leica DMi8; 10 × 10 magnification). Scale bar: 250 μm. (**C**) The morphology of day 7 (D7) differentiated cells was observed using phase-contrast microscopy (Leica DMi8; 10 × 10 magnification). Scale bar: 250 μm. (**D**) HNK1^+^/p75^high^ CNCs were isolated by FACS (BD FACSAria™ Fusion). (**E**) The cell morphology of enriched CNCs during in vitro adherent culture was observed using phase-contrast microscopy (Leica DMi8; 10 × 10 magnification). Scale bar: 250 μm. (**F**) The expression of the neural crest-specific markers including SOX10, p75 and HNK1 in isolated CNCs was detected by immunostaining (LSM 880 with Airyscan; 20 × 10 magnification). Scale bar: 100 μm. (**G**) Relative mRNA expression of pluripotency genes (*OCT4*, *NANOG*), CNC markers (*p75*, *SOX10*, *HNK1*) in CNCs was detected by QRT-PCR; *n* = 3. (**H**) Strategy for deriving pericyte-like cells from CNCs. (**I**) The morphology of pericyte-like cells was detected under phase-contrast microscopy (Leica DMi8; 10 × 10 magnification). Scale bar: 250 μm. (**J**) FACS analysis for the expression surface markers (NG2, PDGFRβ, CD13, and CD146) in CNC PCs (Beckman Coulter CytoFLEX). (**K**) Relative mRNA expression of CNC markers (*p75*, *SOX10*, *HNK1*) and pericyte markers (*NG2*, *PDGFRβ*) in CNC PCs was detected by QRT-PCR; *n* = 3. The data in each panel represent the means ± SD; *p* values were obtained by Student’s two-tailed unpaired *t* test: ** *p* < 0.01, *** *p* < 0.001.

These results prompt us to hypothesize that the transplantation of exogenous pericytes may represent a potential treatment option for pulpitis. In addition, due to the challenges in the direct acquisition of a large number of pericytes from dental pulp, we attempted to derive pericytes from human pluripotent stem cells (hPSCs) through the intermediate stage of cranial neural crests. hPSCs were first inoculated at a low density in neural crest differentiation medium and allowed to differentiate for 7 days using a protocol we previously established (Figure 5A) [22]. We observed that an obvious change in cell morphology started from day 4, and cells become confluent on day 7 (Figure 5B,C). Then, cranial neural crest cells (CNCs) derived from hPSCs were enriched by FACS though the expression of p75^high^/HNK1^+^ surface markers and could be expanded for 10–15 generations in vitro (Figure 5D,E). The results of immunofluorescence staining showed that most of the isolated cells expressed neural crest-specific markers, *SOX10*, *p75*, and *HNK1* (Figure 5F). Using QRT-PCR detection, we further found that the mRNA expressions of the neural crest-specific markers *SOX10*, *p75*, and *HNK1* were highly upregulated, while the expressions of the endogenous pluripotent markers *OCT4* and *NANOG* were significantly decreased in CNCs compared with undifferentiated hPSCs (Figure 5G). These results suggest that CNCs were successfully obtained through our induction protocol.

PDGF-b/PDGFRβ signaling has been reported to have an essential role in pericyte development [7]. P75^high^/HNK1^+^ CNCs enriched by FACS were cultured in pericyte medium containing platelet-derived growth factor BB (PDGFBB) for about 14 days (Figure 5H). During 2 weeks of induction, the CNCs gradually displayed an elongated mesenchymal morphology and exhibited a parallel or spiral arrangement, and thus were referred to as pericyte-like cells (Figure 5I). In order to characterize these pericyte-like cells, we performed FACS analysis, and the results revealed that most (>95%) of the pericyte-like cells expressed pericyte-specific markers, including *NG2*, *PDGFRβ*, *CD13*, and *CD146* (Figure 5J). QRT-PCR analyses were also carried out, and the results showed that the mRNA levels of pericyte markers such as *NG2* and *PDGFRβ* were significantly upregulated, while the expressions of the neural crest-specific markers *SOX10*, *p75*, and *HNK1* were significantly decreased in pericyte-like cells compared with CNCs (Figure 5K). These results indicated that pericytes were successfully generated from hPSC-CNCs using the induction medium containing PDGFBB, and thus were referred to as CNC PCs.

### 3.7. hPSC-Derived Pericytes Have the Ability to Undergo Multilineage Differentiation, Regulate the Immune Response, and Stimulate Angiogenesis

**Figure 6:**
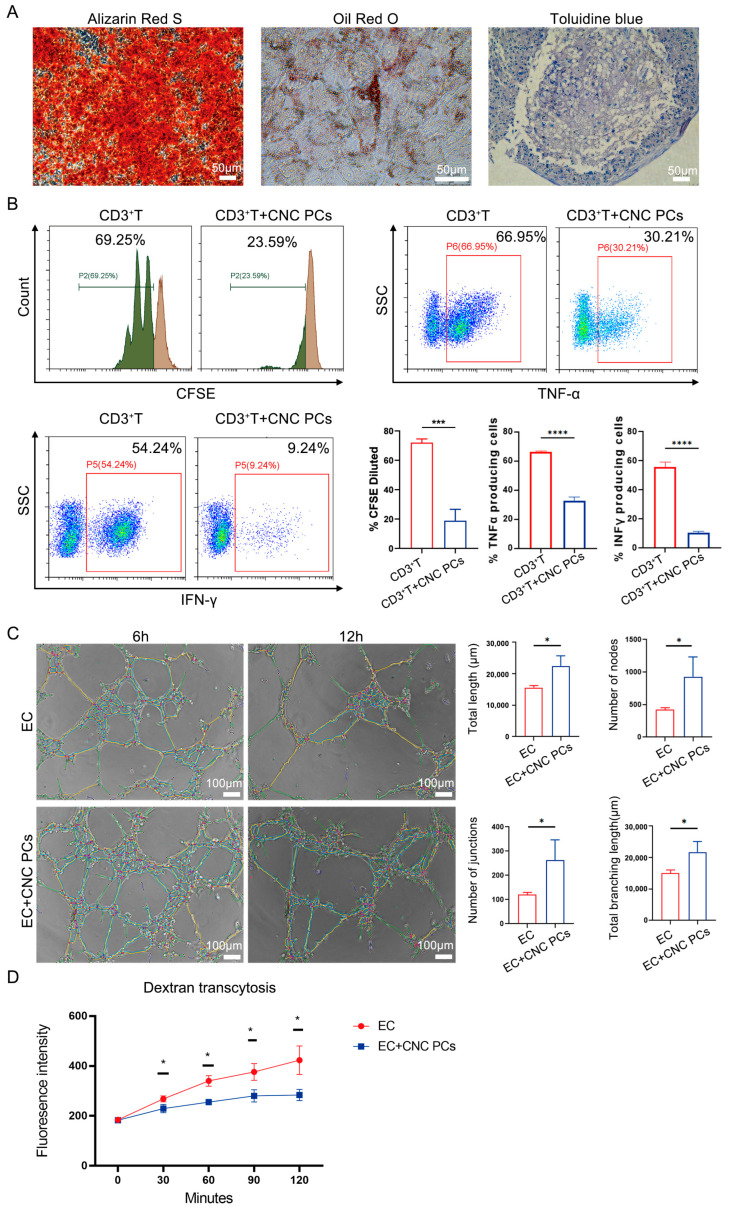
Functional identification of hPSC-derived pericytes in vitro. (**A**) The osteogenic, adipogenic, and chondrogenic differentiation potentials of CNC PCs were verified by Alizarin Red S staining, Oil Red O staining, and Toluidine Blue staining, respectively, and the results were observed under phase-contrast microscope (Leica DMi8; 20 × 10 magnification). Scale bar: 50 μm. (**B**) The effects of CNC PCs on the proliferation (The green color represents divided cells and the brown color represents undivided cells) and proinflammatory cytokine (TNF-α and IFN-γ) production of CD3^+^ T cells were detected by FACS (Beckman Coulter CytoFLEX). *n* ≥ 3. (**C**) Phase-contrast images at 6 and 12 h in tube formation assay. The images captured at 6 and 12 h were used to perform analysis with the angiogenesis plug-in for FIJI and the results of the analysis were overlaid with the bright field images. Four parameters of the angiogenesis analyzer were reported as a measurement of tube forming ability: tube length, number of nodes, number of junctions, and total branching length (Leica DMi8; 10 × 10 magnification); *n* = 3. Scale bar: 100 µm. (**D**) The fluorescence intensity of Dextran in the medium during transcytosis assay was detected; *n* = 3. The data in each panel represent the means ± SD; *p* values were obtained by Student’s two-tailed unpaired *t* test: * *p* < 0.05, *** *p* < 0.001, **** *p* < 0.0001.

Previous studies showed that dental pulp pericytes possess odontogenic differentiation abilities and immunoregulatory abilities [13,31]. To detect the multilineage differentiation potential of hPSC-derived pericytes, CNC PCs were cultured in osteogenic, adipogenic, and chondrogenic induction media for 2–4 weeks. The results of Alizarin Red S, Oil Red O, and Toluidine Blue staining showed that CNC PCs possessed strong osteogenesis and chondrogenesis abilities but relatively weak adipogenesis capacity (Figure 6A). Moreover, to investigate whether the NCSC-derived pericytes had immunoregulatory abilities, we detected the effects of hPSC-derived pericytes on the proliferation and proinflammatory cytokine production of CD3^+^ T cells. The results showed that hPSC-derived pericytes significantly inhibited the proliferation of CD3^+^ T cells (72.09 ± 1.23% for control; 18.89 ± 3.88% for hPSC-derived pericytes; *p* < 0.001) and suppressed the percentages of tumor necrosis factor α (TNF-α) (66.38 ± 0.52% for control; 32.82 ± 2.57% for hPSC-derived pericytes; *p* < 0.0001) and interferon γ (IFN-γ) (55.53 ± 3.46% for control; 10.34 ± 0.97% for hPSC-derived pericytes; *p* < 0.0001) production in CD3^+^ T cells (Figure 6B).

Pericytes are also thought to have the ability to promote angiogenesis and enhance the barrier function of endothelial cells in vivo [7,32]. To verify whether the hPSC-derived pericytes possessed angiogenesis-promoting activity, CNC PCs were first cocultured with human umbilical endothelial cells (EC + CNC PCs group) on Matrigel, and their in vitro tube formation ability was evaluated. Then, 6 or 12 h after coculture, tube networks (vascular-like structure) were observed under the microscope. Interestingly, the CNC PCs had a significantly profound effect on the tube formation of HUVECs compared to HUVECs alone (EC group) by increasing the total length (EC group: 15,552 ± 706.9 μm; EC + CNC PCs group: 22,468 ± 3237.0 μm) and the number of nodes (EC group: 422.3 ± 30.2; EC + CNC PCs group: 928.3 ± 303.8), junctions (EC group: 121.0 ± 8.5; EC + CNC PCs group: 262.0 ± 84.7), and the total branching length (EC group: 15,092 ± 968.3 μm; EC + CNC PCs group: 21,730 ± 3391.0 μm) during tube formation (Figure 6C). In addition, compared with the HUVEC monolayer group (EC group), coculturing with CNC PCs (EC + CNC PCs group) significantly decreased the Alexa 488-dextran fluorescence intensity in the medium in the transcytosis assay (0 min: EC group: 184.3 ± 6.66; EC + CNC PCs group: 182.7 ± 5.86; 30 min: EC group: 268.0 ± 12.49; EC + CNC PCs group: 229.0 ± 16.64; 60 min: EC group: 340.0 ± 21.21; EC + CNC PCs group: 255.3 ± 11.24; 90 min: EC group: 376.3 ± 33.56; EC + CNC PCs group: 280.0 ± 24.56; 120 min: EC group: 423.0 ± 57.42; EC + CNC PCs group: 283.7 ± 22.74) (Figure 6D). Collectively, these results demonstrated that CNC PCs could significantly enhance the angiogenesis capacity and endothelial barrier function of HUVECs and possess strong immunomodulation potential in vitro.

### 3.8. Transplantation of Pericytes Alleviates the Inflammation and Promotes the Regeneration of Dental Pulp

**Figure 7:**
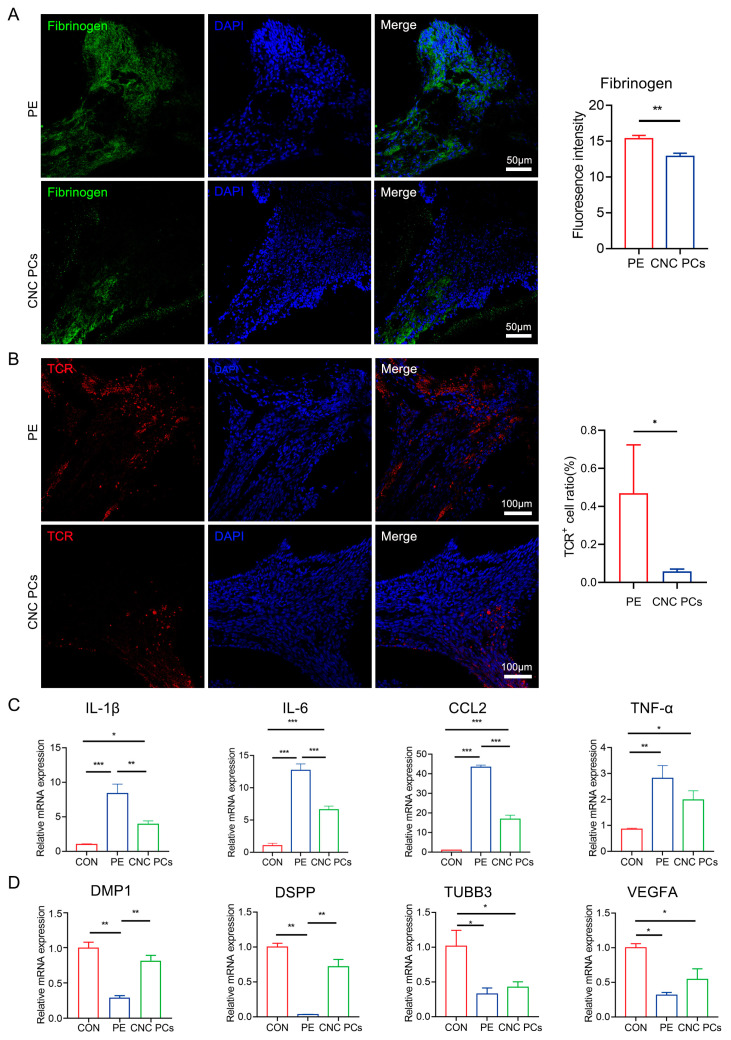
Evaluation of the therapeutic effects of pericyte transplantation in pulpal inflammation. (**A**) Fibrinogen deposits were detected by immunofluorescence staining and compared between pulpal exposure (PE) group and pericyte transplantation (CNC PCs) group (LSM 880 with Airyscan; 20 × 10 magnification); *n* = 3. Scale bar: 50 μm. (**B**) TCR^+^ cells were detected by immunofluorescence staining and compared between pulpal exposure (PE) group and pericyte transplantation (CNC PCs) group (LSM 880 with Airyscan; 20 × 10 magnification); *n* = 3. Scale bar: 50 μm. (**C**) Relative mRNA expression of *IL-1β*, *IL-6*, *CCL2*, and *TNF-α* was detected in pulpal exposure (PE) group and pericyte transplantation (CNC PCs) group; *n* = 3. (**D**) Relative mRNA expression of *DMP1*, *DSPP*, *TUBB3*, and *VEGFA* was detected in pulpal exposure (PE) group and pericyte transplantation (CNC PCs) group; *n* = 3. The data in each panel represent the means ± SD. Student’s two-tailed unpaired *t* test was used in (**A**,**B**). One-way ANOVA analysis was used to compare multiple sets of data in (**C**,**D**); * *p* < 0.05, ** *p* < 0.01, *** *p* < 0.001.

To evaluate the in vivo therapeutic effect of pericytes, CNC PCs were transplanted into the pulp cavities of pulpitis model mice through local injection. First, we detected whether pericytes could survive in inflamed dental pulp in vivo. When DsRedE2-positive CNC PCs were transplanted into the pulp cavity 24 h after pulpal exposure, we showed that DsRedE2^+^ cells could survive in the dental pulp of mice for at least 72 h (Figure S3). To further determine the in vivo therapeutic potential of hPSC-derived pericytes, CNC PCs were locally injected into the pulp cavity 24 h after pulpal exposure, and samples from the untreated control group (pulpal exposure group; PE) and the cell transplantation group (CNC PC group) were collected and analyzed 48 h after pericyte transplantation. To evaluate vascular leakage after the in situ injection of pericytes, fibrinogen staining was performed on pulp tissues. The results showed that the fibrinogen deposits were radically decreased after pericyte transplantation compared with those of the untransplanted group (fluorescence intensity—PE group: 15.34 ± 0.47; CNC PCs group: 12.88 ± 0.44) (Figure 7A), indicating the enhanced vascular barrier function exerted by implanted CNC PCs. We also detected a smaller number of TCR^+^ cells in the pericyte group (0.06 ± 0.01%) than that in the untreated group (0.44 ± 0.25%) (Figure 7B), which may be attributed to the immunoregulatory activity of CNC PCs. In addition, the results of QRT-PCR showed that the expressions of IL-1β, IL-6, and CCL2 were markedly downregulated in the pericyte treatment group compared with those in the untreated group. We also observed a trend of downregulation in the mRNA levels of TNF-α after pericyte transplantation (Figure 7C). More importantly, we found that the expression levels of odontogenic markers including DMP1 and DSPP were significantly rescued, while the mRNA levels of the neural marker TUBB3 and the vascular marker VEGFA showed a mild increase 48 h after pericyte transplantation compared with the control group (Figure 7D). These data indicate that the transplantation of pericytes could alleviate pulpitis, strengthen vascular barrier function, and promote the regeneration of dentin.

### 3.9. LTBP1 Knockdown Considerably Restrains the Immunoregulation Ability of CNC PCs

**Figure 8:**
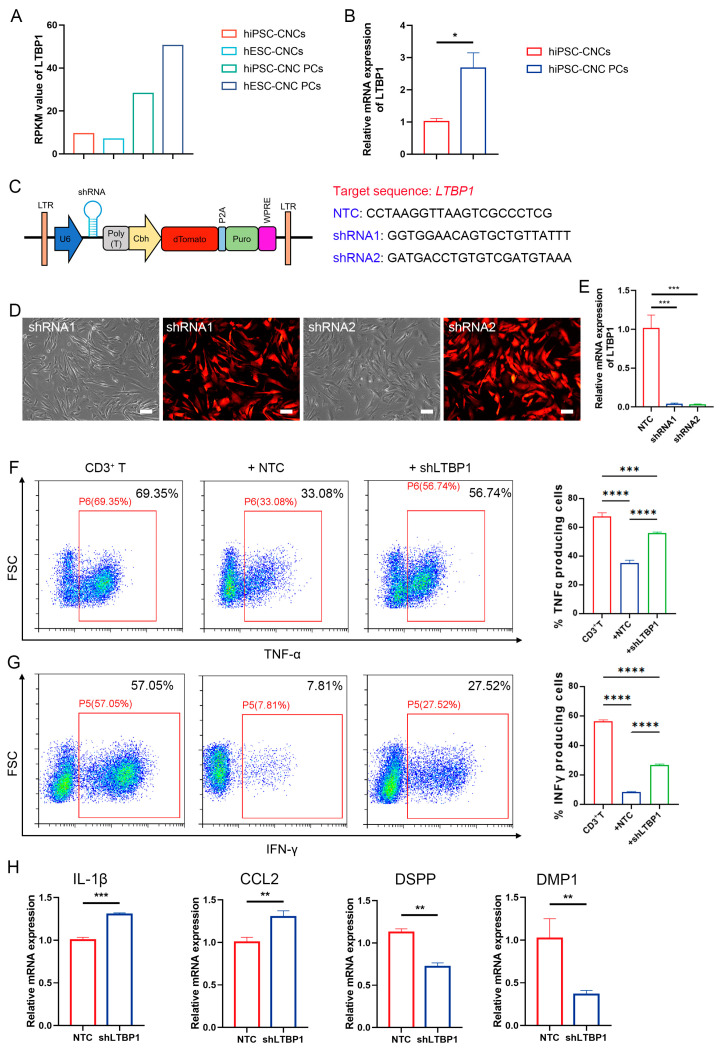
Detection of the role of *LTBP1* in immunoregulatory ability of pericytes. (**A**) The RPKM value of *LTBP1* in hPSC-derived neural crest (hiPSC-CNCs and hESC-CNCs) and pericytes (hiPSC-CNC PCs and hESC-CNC PCs) in RNA-Seq data. (**B**) Detection of *LTBP1* expression in hiPSC-CNCs and hiPSC-CNC PCs by QRT-PCR analysis; *n* = 3. (**C**) The diagram for the shRNA lentiviral vector and the sequences targeting human *LTBP1*. (**D**) CNC PCs transduced with *LTBP1* shRNA lentivirus were observed under phase-contrast and fluorescence microscopy, respectively (Leica DMi8; 20 × 10 magnification). Scale bar: 100 μm. (**E**) The mRNA levels of *LTBP1* before and after *LTBP1* knockdown were detected by QRT-PCR; *n* = 3. (**F**) The effects of CNC PCs on the production of TNF-α from CD3^+^ T cells before or after *LTBP1* knockdown were detected by FACS (Beckman Coulter CytoFLEX); *n* ≥ 3. (**G**) The effects of CNC PCs on the production of IFN-γ from CD3^+^ T cells before or after *LTBP1* knockdown were detected by FACS (Beckman Coulter CytoFLEX); *n* ≥ 3. (**H**) Relative mRNA expression of *IL-1β*, *CCL2*, *DMP1*, and *DSPP* was detected by QRT-PCR and compared between NTC and *LTBP1* knockdown group after pericyte transplantation in pulpitis model; *n* = 3. The data in each panel represent the means ± SD. Student’s two-tailed unpaired *t* test was used in (**B**,**H**). One-way ANOVA analysis was used to compare multiple sets of data in (**E**–**G**); * *p* < 0.05, ** *p* < 0.01, *** *p* < 0.001, **** *p* < 0.0001.

To determine the potential molecules involved in the immunomodulation activity of pericytes, we reanalyzed the RNA-Seq data prepared from hPSC-CNCs and hPSC-CNC PCs (GSE132857) in our previous report [22]. Interestingly, we found that latent transforming growth factor beta binding protein 1 (*LTBP1*) was highly expressed in pericytes (CNC PCs) compared with neural crests (hPSC-CNCs), which was consistent with the results from the QRT-PCR assay (Figure 8A,B). It is known that *LTBP1* plays essential roles in TGFβ function by regulating the folding, secretion, matrix localization, and activation of TGFβ [33,34], but its role in pericyte function is yet to be defined. We then tried to discover whether *LTBP1* is involved in the immunomodulatory activity of pericytes. First, we used an RNA interference tool to knock down the expression of *LTBP1* in pericytes. Short-hairpin RNAs (shRNAs) targeting *LTBP1*, including shRNA1 and shRNA2, were designed and separately integrated into the lentiviral vector carrying a Cbh promoter-driven dTomato construct (Figure 8C). Two days after lentivirus infection, we observed that most of the CNC PCs were dTomato positive (Figure 8D). QRT-PCR assay confirmed that both shRNA1 and shRNA2 efficiently suppressed the mRNA expression of *LTBP1* compared with the nontargeting shRNA (NTC) group (Figure 8E). Then, shRNA1-infected pericytes (designated as sh*LTBP1*) were selected for further analysis.

Then, an in vitro coculture with CD3^+^ T cells was performed. As expected, the results showed that sh*LTBP1* pericytes possessed remarkably inferior immunoregulatory abilities compared to the NTC group in suppressing the T cell secretion of proinflammatory cytokines, including TNF-α (67.42 ± 2.71% for CD3^+^T control group, 35.18 ± 1.94% for CD3^+^T + NTC group, and 56.08 ± 0.68% for CD3^+^T + sh*LTBP1* group) and IFN-γ (56.55 ± 0.78% for CD3^+^T control group, 8.23 ± 0.48% for CD3^+^T + NTC group, and 26.68 ± 0.74% for CD3^+^T + sh*LTBP1* group) (Figure 8F,G). These results strongly indicated the involvement of *LTBP1* in the immunoregulatory properties of pericytes. Moreover, we tried to determine whether the knockdown of *LTBP1* could influence the in vivo regenerative potential of pericytes in the pulpitis model. Forty-eight hours after the transplantation of shRNA1-infected pericytes, samples were collected and the QRT-PCR revealed that the mRNA transcripts of inflammatory factors *IL-1β* and *CCL2* were considerably upregulated, while the expression of odontogenic markers including *DMP1* and *DSPP* were notably decreased in the sh*LTBP1* group compared with that in the NTC group (Figure 8H). These data indicated that the knockdown of *LTBP1* could substantially compromise the immunoregulatory property of pericytes in vivo; this finding was in accordance with the results of the in vitro assay.

## 4. Discussion

One of the major challenges when studying the ontogeny and the differentiation capacity of pericytes is the lack of a single, highly specific marker to identify pericytes [35]. A set of markers has commonly been used to define pericytes, including Pdgfrβ, Desmin, NG2, RGS5, 3G5, CD13, and αSMA [36,37]. The lineage tracing of dental pulp pericytes has been successfully achieved using several transgenic mice models, such as *NG2-CreERT2*, *Fabp4-lacZ*, and *Tagln-Cre* [13,31]. Here, our results showed that, similar to CD13 and NG2, Pdgfrβ could be used as a suitable marker for the labeling of pericytes in dental pulp. We also revealed that the removal of Pdgfrβ+ pericytes induced pulpitis-like pathological features in mice. Nonetheless, it remains unclear whether dental pulp pericyte loss would occur in the presence of bacterial infection in caries or pulpitis. Although the single-cell transcriptome profiling of human dental pulp from normal teeth, enamel caries, and deep dentine caries has been reported [30], changes in pericytes in these pathological conditions were not analyzed in this study. Moreover, no such data from human pulpitis have been reported yet. By reanalyzing these single-cell transcriptome data, we discovered that the percentage of dental pulp pericytes was significantly reduced in the presence of deep caries, suggesting that pericytes were damaged during bacterial infection. Similar pericyte loss was also detected in the mouse model of pulpitis. These data underscore the pivotal role of pericytes in dental pulp homeostasis and further actuate us to verify whether the transplantation of pericytes could alleviate pulp inflammation and promote pulp regeneration, which has not been addressed to date.

The isolation of pericytes directly from dental pulp is hindered by their low frequency, relatively limited proliferative capacity, and potential microbial contamination [38]. hPSCs have the potential for long-term self-renewal and differentiation into almost all cell types in vitro, and thus represent an ideal cell source for the derivation of pericytes [20,39]. Therefore, we tried to generate pericytes from hPSCs. To achieve this, we tried to determine the developmental origin of dental pulp pericytes. It was reported that pericytes in all blood vessels in the face and forebrain are derived from the cephalic (cranial) neural crest [29]. A previous study also detected high expression levels of neural crest markers Snai1 and Snai2 in dental pulp pericytes in mice, further suggesting the neural crest origin of dental pulp pericytes [31]. Here, using the neural crest lineage-tracing model *Wnt1-Cre2:Rosa26-tdTomato*, we found that almost all of the Pdgfrβ^+^ pericytes were positive for tdTomato, which further confirmed that these dental pulp pericytes were indeed neural crest derivatives. Based on these results, we employed a two-step induction protocol for pericyte derivation from hPSCs through the intermediate stage of cranial neural crests. Undifferentiated hPSCs were treated with CHIR99021 and SB431542 for 7 days to generate p75^high^/HNK1^+^ migrating cranial neural crests, which were further isolated using FACS and cultured in pericyte medium for 2 weeks; then, PDGFRβ^+^/CD13^+^/NG2^+^/CD146^+^ pericytes were successfully generated. Collectively, our protocol supplies an easy, fast, and effective method for the derivation of pericytes with cranial neural crest origin from hPSCs.

Pericytes are thought to mainly be involved in vascular maintenance in different tissues [7]. Indeed, our results showed that hPSC-derived pericytes markedly enhanced the in vitro tube formation ability of HUVECs. Previous studies also showed that NG2^+^ pericytes in dental pulp have the ability to differentiate into odontoblasts [13]. Moreover, through bulk RNA-seq and single cell RNA-seq analysis, Val Yianni and Paul T. Sharpe [31] discovered that mouse pericytes have an immunomodulatory role as they express multiple cytokines and chemokines within the dental pulp. Here, we demonstrated that pericytes derived from hPSCs possessed multipotency and could be induced to osteogenic, chondrogenic, and adipogenic cell lineages. More importantly, we presented direct evidence that hPSC-derived pericytes could efficiently inhibit the proliferation and expression of inflammatory factors TNF-α and IFN-γ in CD3^+^ T cells. These results suggest that pericytes generated from hPSCs in vitro share similar biological characteristics with their in vivo counterparts. These data also reveal that pericytes possess mesenchymal stem cell-like properties, as previously reported [32], and may represent precursors of MSCs in vivo [40,41,42]. Interestingly, Nuno et al. demonstrated that pericytes of multiple organs did not behave as mesenchymal stem cells in vivo [43]. In our previous study, we also discovered that pericytes showed much stronger contraction ability than mesenchymal stem cells in both a gel lattice contraction assay and carbachol treatment assay [22]. This evidence indicates that there are some functional differences between pericytes and mesenchymal stem cells.

RCT is currently the most widely used clinical treatment for pulpitis. Nevertheless, teeth that experience root canal treatment lose vitality and essential functions, resulting in teeth that are nonvital, prone to reinfection, and fragile [44]. Currently, there are data suggesting that pulp capping, a common treatment for VPT, can also induce the formation of restorative dentin bridges [45,46]. However, pulp capping is mainly used for medically induced and traumatic pulp exposures with small perforation diameters. With the rapid advancement in cell biology and tissue engineering techniques, stem cell-based dental pulp regeneration has become a new research focus for clinical therapy. It has been shown that the transplantation of stem cells can produce ectopic pulp-like tissue, indicating a broad prospect for clinical application [47]. Different types of stem cells have been used for dental pulp regeneration with significant therapeutic benefits, such as dental pulp stem cells (DPSCs) [5], dental follicle stem cells (DFSCs) [48], periodontal ligament stem cells (PDLSCs) [1], and stem cells from human exfoliated deciduous teeth (SHEDs) [1]. Previous studies showed that dental pulp stem cells (DPSCs) highly expressed typical perivascular markers, including alpha-smooth muscle actin (α-SMA), NG2, PDGFRβ, 3G5, and CD146 [37]. Nonetheless, lineage-tracing studies revealed that only small populations of newly differentiated odontoblasts are contributed by NG2^+^ perivascular cells, while most are derived from other MSC-like cells of nonpericyte origin in the dental pulp [49,50]. Recently, single-cell RNA sequencing data regarding freshly isolated and monolayer cultured human DPSCs were reported. In this study, 14 cell clusters were identified, while only cells in cluster 5 highly expressed pericyte markers, such as NG2, PDGFRβ, NOTCH3, and ACTA2 [51]. These data suggest that dental pulp pericytes share similar characteristics but are not identical to DPSCs. In addition, our results demonstrate that the barrier function of pericytes is vital for dental pulp homeostasis maintenance, since the loss of pericytes induces vascular leakage, the infiltration of immune cells, and damage to the odontoblast cell layer. Most importantly, for the first time, we showed that pericyte transplantation could efficiently restore the vascular barrier function, suppress pulpal inflammation, and promote the regeneration of dentin. In addition, it is known that neural substrates play crucial roles in maintenance of tissue homeostasis, and their dysfunction is involved in the pathogenesis of different types of diseases, including neurological and psychiatric disorders as well as pulpitis [5,52,53]. One previous study showed that transplantation of deciduous autologous DPSCs efficiently promoted the regeneration of pulp tissues including nerves in patients with pulp necrosis [5]. Here, we also observed a trend (although not statistically significant) in the recovery of the mRNA level of the pan-neural marker *TUBB3* when treated with CNC PCs in pulpitis model mice, which needs to be further verified by long-term experimental observation. These results suggest that pericytes display similar regenerative capability to DPSCs in vivo.

*LTBP1* is an extracellular matrix protein and is abundantly expressed by cells in the heart, lungs, and other tissues [54,55,56]. The knockout of *LTBP1* in mice could lead to cardiovascular defects, craniofacial abnormalities, and the shortening of long bones [57]. Katri et al. also showed that *LTBP1* is implicated in the proliferation and osteogenic differentiation of human mesenchymal stem cells [58]. Further research revealed that *LTBP1* is part of a large latent complex with TGFβ and its propeptide, and it plays important roles in TGFβ1 secretion and activation [54,58]. Additionally, TGFβ1 derived from human mesenchymal stem cells is reported to possess strong immunosuppressive potential in inhibiting inflammatory factor secretion from anti-CD3/CD28-stimulated splenocytes [59]. Nevertheless, whether *LTBP1* is involved in the immunomodulation ability of mesenchymal stem cells or pericytes has not yet been addressed. Interestingly, our results indicated an immunosuppressive characteristic of *LTBP1* in pericytes. These results, however, were contradicted with one recently published study, which illustrated that the downregulation of *LTBP1* expression could activate the immunosuppressive signaling pathway in the tumor microenvironment of a cervical cancer model [60], which could be due to the differences in the cell types studied. In addition, the underlying molecular mechanism of *LTBP1* in the immunomodulatory capacity of pericytes needs to be clarified further.

## 5. Conclusions

In this study, we found that the loss of pericytes could induce pulpitis-like phenotypes in the uninfected dental pulp of mice, indicating an important role of pericytes in homeostasis maintenance in dental pulp. Our results also revealed that pericytes were greatly reduced during inflammation, including in deep dental caries and pulpitis. More importantly, for the first time, we showed that the implantation of hPSC-derived pericytes could effectively inhibit pulp inflammation and promote the regeneration of dentin, which was partially contributed by *LTBP1*-mediated T cell suppression. However, the present study had several limitations. First, the optimal timing of pericyte administration was not determined, because pulpitis has different stages of disease onset. Second, the long-term treatment effect of pericyte transplantation on pulp regeneration needs further investigation. Overall, the transplantation of pericytes may represent a new prospective therapeutic option for patients with pupal inflammation.

## Data Availability

The datasets generated and analyzed during the current study are available from the corresponding author upon reasonable request.

## Supplementary Materials

The following supporting information can be downloaded at: https://www.mdpi.com/article/10.3390/biomedicines11123199/s1, Figure S1: Dental pulp pericyte lineage tracing using *Wnt1-Cre2:Rosa26-tdTomato* mice model; Figure S2: Single-cell transcriptome profiling of human dental pulp from normal teeth, enamel caries, and deep dentine caries; Figure S3: Transplanted DsRedE2^+^ CNC PCs survived for at least 72 h in vivo after transplantation; Table S1: Primers used for QRT-PCR; Table S2: Antibodies used in immunofluorescence staining; Table S3: Antibodies used in FACS.
