# Supplementary material for: Efficient Treatment of Pulpitis via Transplantation of Human Pluripotent Stem Cell-Derived Pericytes Partially through LTBP1-Mediated T Cell Suppression
Source: Biomedicines. 2023 Dec 1;11(12):3199. doi: 10.3390/biomedicines11123199 (PMC10740489; doi:10.3390/biomedicines11123199)
Supplement: Supplementary file 1 [file biomedicines-11-03199-s001.zip › biomedicines-2652370-supplementary.pdf]

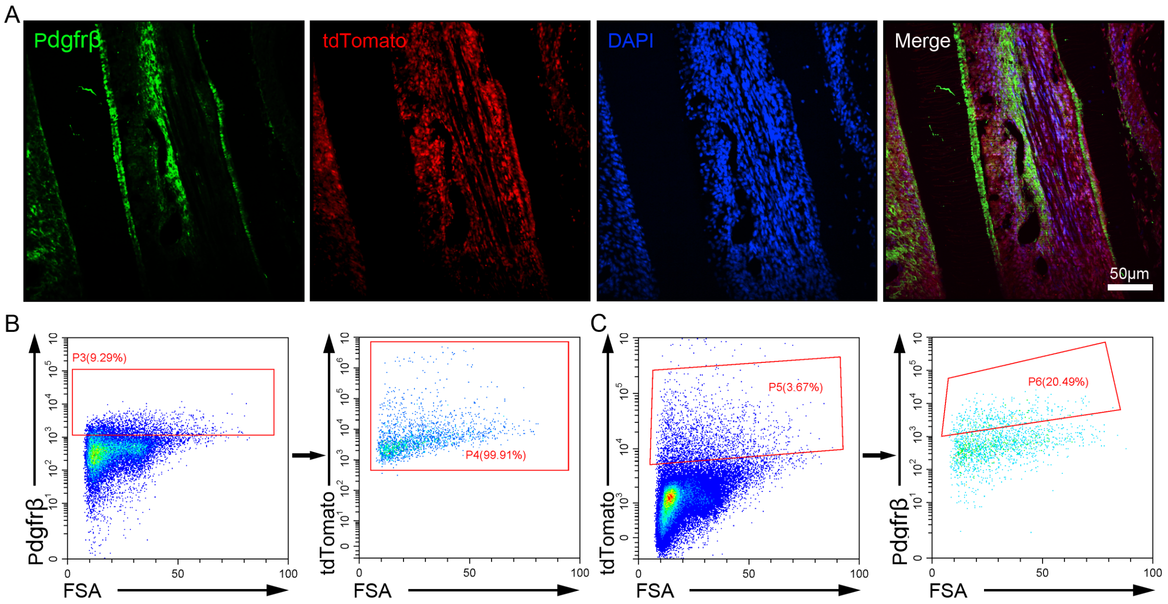

Figure S1. Dental pulp pericyte lineage tracing using *Wnt1-Cre2:Rosa26-tdTomato* mice model.

A. Detection of Pdgfrβ^{+} (green) cells in dental pulp of *Wnt1-Cre2:Rosa26-tdTomato* mice by immunofluorescence staining (LSM 880 with Airyscan; 40 × 10 magnification). Scale bar: 50 μm.

B. FACS analysis for tdTomato expression in dental pulp Pdgfrβ^{+} cells (Beckman Coulter CytoFLEX).

C. FACS analysis for the expression of Pdgfrβ in tdTomato^{+} cells (Beckman Coulter CytoFLEX).

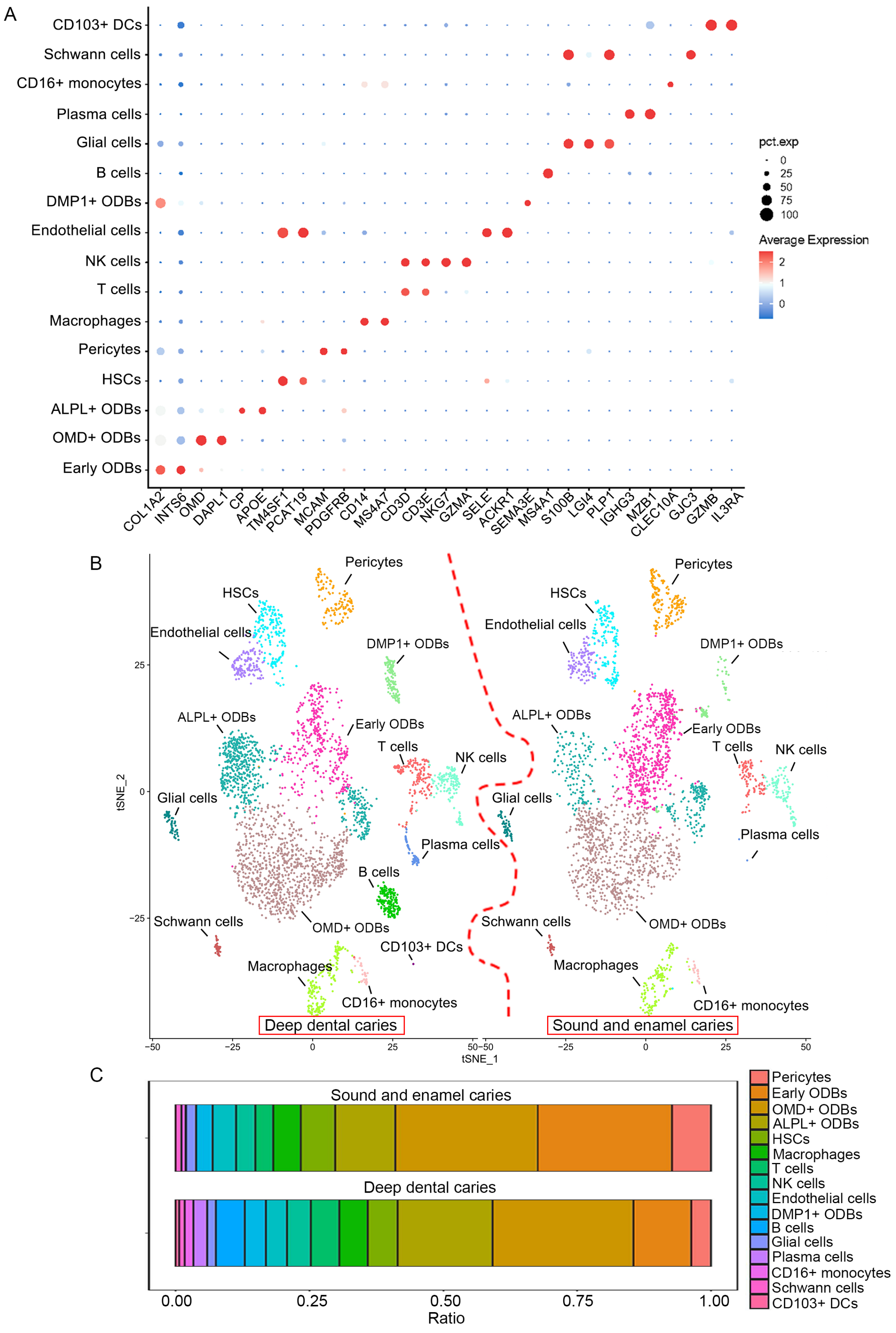

Figure S2. Single-cell transcriptome profiling of human dental pulp from normal teeth, enamel caries, and deep dentine caries.

A. The average expression levels of the marker genes used in the cell-type

annotation.

B. UMAP plots showing different distributions of cell populations in the sound/enamel caries and deep dental caries.

C. Stacked bar plots comparing the percentages of cell populations from the sound/enamel caries and deep dental caries.

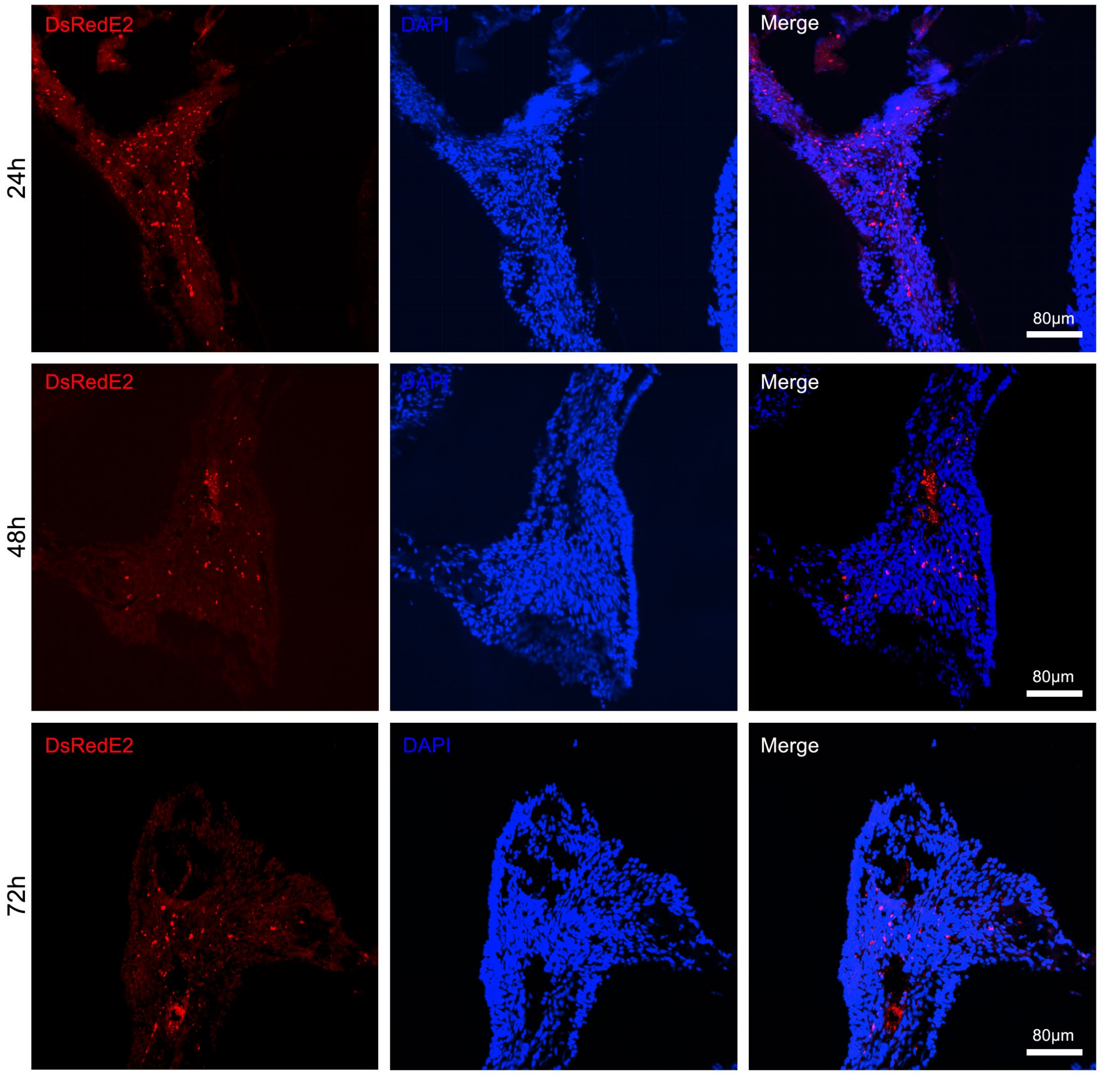

Figure S3. Transplanted DsRedE2$^+$ CNC PCs survived for at least 72 h in vivo after transplantation.

Detection of DsRedE2$^+$ (red) cells in dental pulp by immunofluorescence staining (LSM 880 with Airyscan; 20 × 10 magnification). Scale bar: 80 μm.

Supplementary Table S1. Primers used for QRT-PCR.

Gene	Direction	Sequences
IL-1β	Forward	5′ GAA ATG CCA CCT TTT GAC AGT G 3′
	Reverse	5′ TGG ATG CTC TCA TCA GGA CAG 3′
IL-6	Forward	5′ TAG TCC TTC CTA CCC CAA TTT CC 3′
	Reverse	5′ TTG GTC CTT AGC CAC TCC TTC 3′
TNF-α	Forward	5′ CCC TCA CAC TCA GAT CAT CTT CT 3′
	Reverse	5′ GCT ACG ACG TGG GCT ACA G 3′
CCL2	Forward	5′ TTA AAA ACC TGG ATC GGA ACC AA 3′
	Reverse	5′ GCA TTA GCT TCA GAT TTA CGG GT 3′
MMP9	Forward	5′ GCA GAG GCA TAC TTG TAC CG 3′
	Reverse	5′ TGA TGT TAT GAT GGT CCC ACT TG 3′
DMP1	Forward	5′ CAT TCT CCT TGT GTT CCT TTG GG 3′
	Reverse	5′ TGT GGT CAC TAT TTG CCT GTG 3′
DSPP	Forward	5′ ATT CCG GTT CCC CAG TTA GTA 3′
	Reverse	5′ CTG TTG CTA GTG GTG CTG TT 3′
β-actin	Forward	5′ TAT GGA ATC CTG TG GCA TC 3′
	Reverse	5′ GTG TTG GCA TAG AGG TCT T 3′
GAPDH	Forward	5′ GAA GGT GAA GGT CGG AGT C 3′
	Reverse	5′ GAA GAT GGT GAT GGG ATT TC 3′
OCT4	Forward	5′ GAC AGG GGG AGG GGA GGA GCT AGG 3′

	Reverse	5′ CTT CCC TCC AAC CAG TTG CCC CAA AC 3′
NANOG	Forward	5′ CAG CCC AGA TTC TTC CAC CAG TCC C 3′
	Reverse	5′ CGG AAG CGT TCC CAG TCG GGT TCA CC 3′
p75	Forward	5′ CTG CCT GGA CAG CGT GAC GTT 3′
	Reverse	5′ GCA GCG CCC AGT CGT CTC AT 3′
SOX10	Forward	5′ CCC GCA CTA CAC CGA CCA 3′
	Reverse	5′ AGG AGA AAG CCG AGT AGA 3′
HNK1	Forward	5′ CTC CTT CGA GAA CTT GTC ACC 3′
	Reverse	5′ GGG TCA GTG AAG CCC TTC TT 3′
NG2	Forward	5′ GTC TTT TGA GGC TGC CTG TC 3′
	Reverse	5′ CTG TGT GAC CTG GAA GAG CA 3′
PDGFRβ	Forward	5′ ACC TGC AAT GTG ACG GAG AG 3′
	Reverse	5′ GGG TGC GGT TGT CTT TGA AC 3′
TUBB3	Forward	5′ GCG CCT TTG GAC ACC TAT TCA 3′
	Reverse	5′ GCC CTC CGT ATA GTG CCC T 3′
VEGFA	Forward	5′ GCA CAT AGA GAG AAT GAG CTT CC 3′
	Reverse	5′ CTC CGC TCT GAA CAA GGC T 3′
LTBP1	Forward	5′ CTG ACG GCC ACG AAC TTC C 3′
	Reverse	5′ GCA CTG ACA TTT GTC CCT TGA 3′

Supplementary Table S2. Antibodies used in immunofluorescence staining.

**Antigen**	**Host**	**Dilution**	**Company**	**Cat. No.**
PDGFRβ	mouse	1:20	Santa Cruz	sc-374573
Lycopersicon Esculentum (Tomato) Lectin (LEL, TL), DyLight™ 649		1:50	Vector	DL-1178-1
Fibrinogen	rabbit	1:100	Abcam	ab92572
TCR β		1:100	BD Pharmingen	553172
HNK1	mouse	1:200	Sigma-Aldrich	c6680
SOX10	goat	1:100	R&D System	AF2864
p75	rabbit	1:200	Promega	G3231
Goat anti-mouse IgG Alexa 555	goat	1:1000	Invitrogen	A21422
Goat anti-rabbit IgG Alexa 488	goat	1:1000	Invitrogen	A11008
Goat anti-rabbit IgG Alexa 555	goat	1:1000	Invitrogen	A21428
Donkey anti-goat IgG Alexa 488	donkey	1:1000	Invitrogen	A32814

Supplementary Table S3. Antibodies used in FACS.

Antigen	Label	Company	Cat. No.
Anti-Human CD13	PE	BD Pharmingen	555394
Anti-Human CD146	PE	BD Pharmingen	550315
Anti- Human NG2	Alexa Fluor 647	BD Pharmingen	562414
Anti-Human CD140b	PE	BD Pharmingen	558821
Anti-Human CD271 (p75)	Alexa Fluor 647	BD Pharmingen	560326
Anti-Human CD57 (HNK1)	PE	BD Pharmingen	560844
Anti-Human CD3	V450	BD Pharmingen	560365
Anti-Human TNF	PE	BD Pharmingen	554513
Anti-Human IFN	PE-Cy7	BD Pharmingen	557643
Anti-Mouse CD13	FITC	BD Pharmingen	558744

Anti- Mouse CD140b (Pdgfrβ)	Alexa Fluor 647	BD Pharmingen	558428
